# Comprehensive analysis of the expression and prognosis for RAI2: A promising biomarker in breast cancer

**DOI:** 10.3389/fonc.2023.1134149

**Published:** 2023-03-29

**Authors:** Ying Jiao, Shiyu Li, Juejun Gong, Kun Zheng, Ya Xie

**Affiliations:** ^1^ Department of Oncology, Tongji Hospital of Tongji Medical College, Huazhong University of Science and Technology, Wuhan, China; ^2^ Department of Medicine and Therapeutics, State Key Laboratory of Digestive Disease, Institute of Digestive Disease, Li Ka Shing Institute of Health Sciences, CUHK-Shenzhen Research Institute, The Chinese University of Hong Kong, Hong Kong, Hong Kong SAR, China; ^3^ Department of Oncology, The Central Hospital of Wuhan, Tongji Medical College, Huazhong University of Science and Technology, Wuhan, China; ^4^ Biological Sciences, Faculty of Environmental and Life Sciences, University of Southampton, Southampton, United Kingdom; ^5^ Institute for Life Sciences, University of Southampton, Southampton, United Kingdom; ^6^ Department of Gynecology, The First Affiliated Hospital of Zhengzhou University, Zhengzhou, China

**Keywords:** RAI2, biomarker, breast cancer, prognosis, tumor progression

## Abstract

**Introduction:**

Retinoic acid-induced 2 (RAI2) was initially related to cell differentiation and induced by retinoic acid. RAI2 has been identified as an emerging tumor suppressor in breast cancer and colorectal cancer.

**Methods:**

In this study, we performed systematic analyses of RAI2 in breast cancer. Meta-analysis and Kaplan-Meier survival curves were applied to identify the survival prediction potential of RAI2. Moreover, the association between RAI2 expression and the abundance of six tumor-infiltrating immune cells was investigated by TIMER, including B cells, CD8+ T cells, CD4+ T cells, B cells, dendritic cells, neutrophils, and macrophages. The expression profiles of high and low RAI2 mRNA levels in GSE7390 were compared to identify differentially expressed genes (DEGs) and the biological function of these DEGs was analyzed by R software, which was further proved in GSE7390.

**Results:**

Our results showed that the normal tissues had more RAI2 expression than breast cancer tissues. Patients with high RAI2 expression were related to a favorable prognosis and more immune infiltrates. A total of 209 DEGs and 182 DEGs were identified between the expression profiles of high and low RAI2 mRNA levels in the GSE7390 and GSE21653 databases, respectively. Furthermore, Gene Ontology (GO) enrichment indicated that these DEGs from two datasets were both mainly distributed in “biological processes” (BP), including “organelle fission” and “nuclear division”. Kyoto Encyclopedia of Genes and Genomes (KEGG) pathways analysis demonstrated that these DEGs from two datasets were both significantly enriched in the “cell cycle”. Common hub genes between the DEGs in GSE7390 and GSE21653 were negatively associated with RAI2 expression, including CCNA2, MAD2L1, MELK, CDC20, and CCNB2.

**Discussions:**

These results above suggested that RAI2 might play a pivotal role in preventing the initiation and progression of breast cancer. The present study may contribute to understanding the molecular mechanisms of RAI2 and enriching biomarkers to predict patient prognosis in breast cancer.

## 1 Background

Breast cancer (BRCA), which accounts for approximately 30% of female cancers, is the first leading cause of cancer death for women aged 20 to 59 years (1). Several major molecular subtypes in BRCA are defined by the presence or absence of hormone receptors (estrogen receptors (ER) or progesterone receptors (PR) and human epidermal growth factor receptor 2 (HER2) (2). Over the decades, molecular classification exerts a critical role in predicting tumor behavior and guiding therapeutic strategies (3). Nearly 75% of BRCA patients express hormone receptors, which results in the potent effects of endocrine therapy (2). However, some patients get primary or acquired resistance to endocrine therapy (4). Moreover, approximately 20-30% of BRCAs patients overexpress HER2 and have poorer prognoses with more aggressive tumors (5). Although anti-HER2 treatment has shown some achievements for HER2-positive BRCAs, continuing high mortality of HER2-overexpressed BRCAs demands new therapies (6). Thus, it is crucial to investigate further novel biomarkers and emerging agents to treat BRCA.

Retinoic acid (RA) is one of the metabolic products of retinol (vitamin A) and exerts activity in distinct isometric forms, including 9-cis-RA, 13-cis-RA as well as all-trans-RA (7). Retinoic acid-induced 2 (RAI2) is induced by RA in embryonal carcinoma cells and involved in cellular differentiation (8, 9). Stefan Werner et al. found that RAI2 acts as a transcriptional regulator that contributes to the expression of some critical regulators of breast epithelial integrity (10). Low RAI2 expression was significantly associated with early hematogenous metastasis of BRCA cells to bone marrow, especially in ER^+^ BRCA (10). Moreover, RAI2 inhibited AKT signaling to suppress CRC progression, and methylated RAI2 indicated poor prognosis in CRC (11).

Some studies used invasive breast cancer tissues to indicate that lower RAI2 mRNA and protein were independent risk factors for lower shorter disease-free survival (DFS), and breast-cancer-specific survival (BCSS) (12). However, there are not sufficient studies to explain the biological function of RAI2 in BRCA. Furthermore, more samples are needed to validate the effects of RAI2 on prognosis for breast cancer patients and RAI2-related pathways should be clarified. More investigations on RAI2 might provide novel therapeutic strategies for clinicians (13).

## 2 Materials and methods

### 2.1 Search strategy of the GEO datasets

The datasets of human BRCA with RAI2 mRNA expression were searched in the GEO databases (https://www.ncbi.nlm.nih.gov/geo/), up to December 10, 2015. The search terms “breast cancer” (“breast neoplasm,” “breast tumor,” “breast carcinoma,” and “mammary cancer”) were used and the search scope was based on homo sapiens. Only the databases that met the inclusion criteria were chosen in the following analysis.

### 2.2 Inclusion criteria

Based on the PRISMA statement criteria, we chose databases that matched the following inclusion criteria: 1) the databases were about BRCA or normal breast tissues, and homo sapience rather than cell lines; 2) the datasets were about mRNA rather than DNA or microRNA and included RAI2 mRNA expression; 3) the sample capacity was more than 45; 4) it was available for clinic-pathological and prognosis information of BRCA patients in these databases, such as T stage, N stage, TNM stage, grade, molecular subtypes, and clinical outcome. 5) when several databases shared specific patient identification, we chose the latest and most complete datasets.

### 2.3 Data extraction

We performed in silico validation of RAI2, using 30 publicly available breast datasets with 4863 samples. Data analysis and technical graphics were performed independently by two individuals. We extracted data from every dataset to a predefined table with a standardized data collection form, with information recorded as follows: first author’s name, publication year, country or area, follow-up duration, tumor stage, patient number, detection methods, and platform. The median of RAI2 mRNA expression was used for cutoff values. The probe with the maximum mRNA expression value was extracted while there was more than one probe for RAI2. Overall survival (OS), metastasis-free survival (MFS), and relapse-free survival (RFS) were evaluated by Cox proportional hazard ratio (HR) and 95% confidence interval (CI) using the extracted data. These data were shown in **Supplemental Table 1**.

### 2.4 Meta-analysis

The meta-analysis was performed using the STATA software (Version 12.0; StataCorp LP, College Station, TX, USA). The heterogeneity of publication was assessed by the inconsistency index (*I* ^2^) statistic. The random-effect model was employed on the condition that the *I* ^2^ value was more than 50%, which indicated that heterogeneity was shown. The fixed-effect model was used on the condition that the *I* ^2^ value was less than 50%, which demonstrated that homogeneity was presented. The publication bias was evaluated by Begg’s and Egger’s tests. To evaluate the association between RAI2 expression level and clinicopathological parameters, the odds ratio (OR) and its corresponding 95% CI were calculated. To evaluate the effects of RAI2 expression level on the survival outcome of BRCAs, we assessed the hazard ratio (HR) and its corresponding 95% CI. HR > 1 indicated that higher expression of RAI2 predicted worse survival.

### 2.5 Survival analysis

We performed Kaplan-Meier survival curves with HR and log-rank *p*-value for the RAI2 mRNA expression level of BRCAs by the online analysis tool (http://www.kmplot.com/analysis/). The Affymetrix probe ID for RAI2 was 219440_at. The patients were divided into two groups according to the median expression value. We chose 120 months as the follow-up threshold. Every intrinsic subtype of BRCA was conducted, including luminal A, luminal B, HER2^+^, and basal-like types.

### 2.6 Analysis of TIMER datasets

The online tool, Tumor Immune Estimation Resource (TIMER, https://cistrome.shinyapps.io/timer/) included RNA-seq profiles of 10897 samples from 32 cancers in The Cancer Genome Atlas (TCGA) (14). The association of RAI2 expression with the abundance of six tumor-infiltrating immune cells could be evaluated based on silico deconvolution approaches *via* gene module in TIMER, including CD8+ T cells, CD4+ T cells, B cells, dendritic cells, neutrophils, and macrophages. The results were demonstrated in scatterplots with *p*-value and purity-corrected partial Spearman’s correlation (partial-cor).

### 2.7 Identification of DEGs

Based on the databases of GSE7390 and GSE21653, we compared the two expression profiles of high and low RAI2 mRNA expression in BRCAs, which were divided according to the median expression by R software (version 3.6.3; https://www.r-project.org/) to identify DEGs. The results were obtained using limma package (version 3.42.2) (15) and pheatmap package (version 1.0.12). The limma package was employed to determine DEGs with log |fold change| > 1 and adj. *p* < 0.05 in unpaired t-test as cut-off levels. Subsequently, we used pheatmap package to generate a heatmap, demonstrating the differential expression levels of the top 40 DEGs directly, and a volcano plot was produced to show all DEGs in this dataset.

### 2.8 GO enrichment and KEGG pathways analysis

To discover the biological functions of DEGs, the GO enrichment, and KEGG pathways analysis were performed by R software (16, 17). Colorspace package (version 1.4-1), stringi package (version 1.4.6), ggplot2 package (version 3.3.0), and Bioconductor packages, such as DOSE package (version 3.12.0) (18), clusterProfiler package (version 3.14.3) (19) and enrichplot package (version 1.6.1), were used to get bubble plot and barplot. *p* < 0.05 was statistically significant.

### 2.9 PPI network construction

The PPIs of the DEGs were identified using the online Search Tool for Retrieval of Interacting Genes (STRING) database (version 11.0). All DEGs were mapped into this database to evaluate the interactive relations, showing nodes with the confidence score >0.4 and hiding disconnected nodes in the network. Subsequently, the Cytoscape software (version 3.7.2; National Resource for Network Biology) was employed to get PPI networks (20). The criteria for disease module searching were set as follows: Molecular COmplex DEtection (MCODE) score > 3, and each module must have more than four nodes. *p* < 0.05 was considered a statistically significant difference. Besides, the top 10 hub genes ranking by degree were identified using cytoHubba application on Cytoscape with the shortest path displaying (21).

### 2.10 The relation between RAI2 and several genes

The correlation of RAI2 and several hub genes were shown by GraphPad Prism (version 8.0.1). We chose the datasets of GSE7390 with 198 primary BRCA patients and GSE21653 with 266 patients to present the relation between RAI2 and hub genes, including CCNA2, CDC20, CDC6, MAD2L1, TTK, MELK, and CCNB2.

## 3 Results

### 3.1 RAI2 expression relates to the molecular subtypes of BRCA

**Figure 1:**
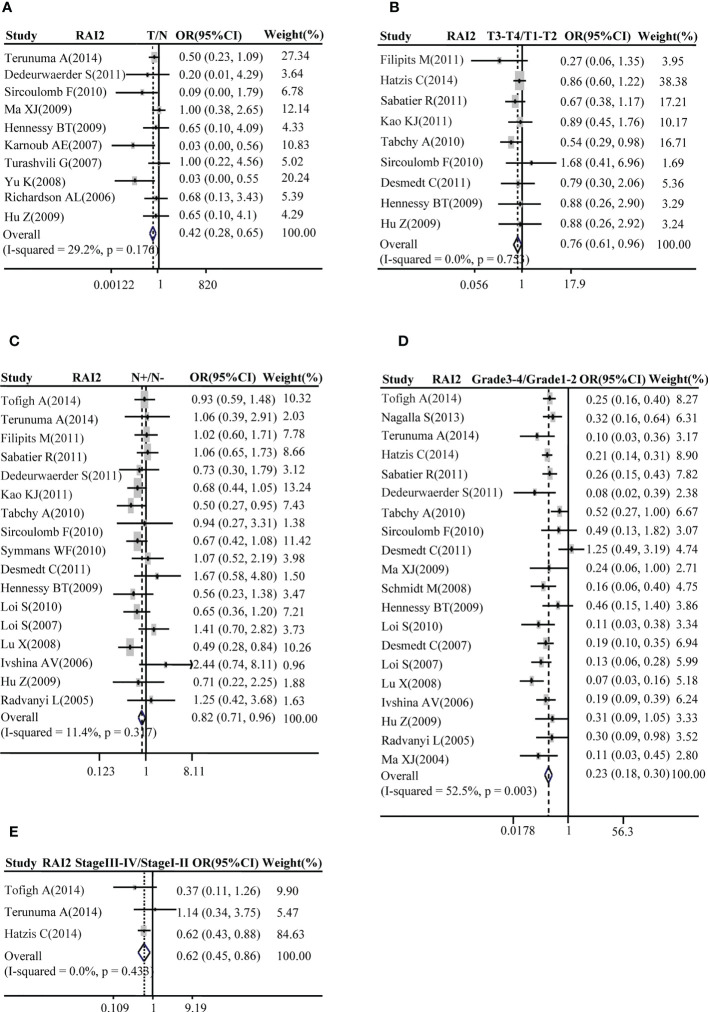
Correlation between RAI2 mRNA expression and breast cancer as evaluated by the OR. Relation of RAI2 mRNA expression with breast cancer risk compared with normal breast tissues **(A)**. Association between RAI2 mRNA expression and breast cancer risk compared with T stage **(B)**, N status **(C)**, histological grade **(D)**, and clinical TNM stage **(E)**. RAI2, retinoic acid-induced 2; CI, confidence interval; OR, odds ratio; TNM, tumor–node–metastasis.

A total of ten studies with GEO datasets indicated that RAI2 mRNA expression in BRCA samples was reduced when compared with healthy control samples (pooled OR: 0.42, 95% CI: 0.28-0.65; Cochran’s *Q* test *p* = 0.176 and *I* ^2^ = 29.2%; **Figure 1A**). Then, some studies were adopted to further evaluate the relation between RAI2 mRNA expression and clinicopathological features of BRCA. The results showed that RAI2 mRNA expression in mammary cancer had a negative relation with T stage (pooled OR: 0.76, 95% CI: 0.61-0.96; Cochran’s Q test, *p* = 0.753 and *I^2^* = 0.0%; **Figure 1B**), N status (pooled OR: 0.82, 95% CI: 0.71-0.96; Cochran’s Q test, *p* = 0.317 and *I^2^* = 11.4%; **Figure 1C**), histological grade (pooled OR: 0.23, 95% CI: 0.18-0.30; Cochran’s Q test, *p* = 0.003 and *I^2^* = 52.5%; **Figure 1D**) and TNM stage (pooled OR: 0.62, 95% CI: 0.45-0.86; Cochran’s Q test, *p* = 0.433 and *I^2^* = 0.0%; **Figure 1E**).

**Figure 2:**
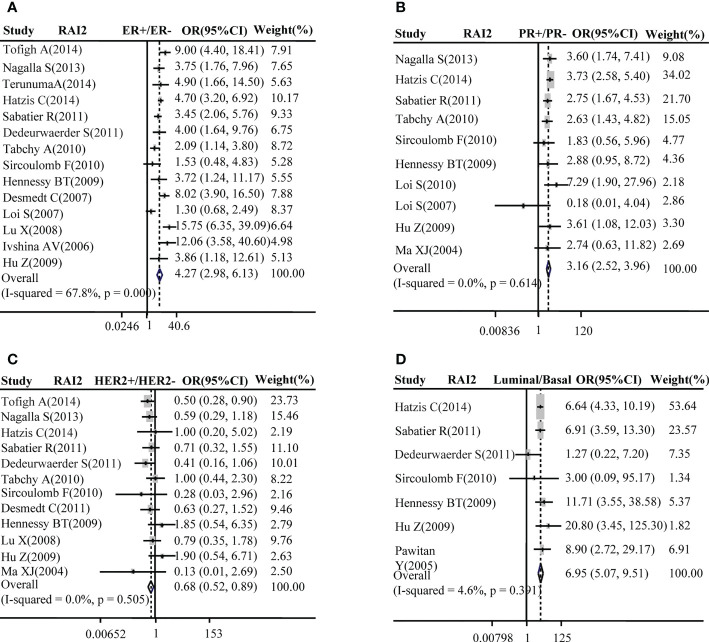
Correlation between RAI2 mRNA expression and molecular subtype. Correlation between RAI2 mRNA expression with ER status **(A)**, PR **(B)**, HER2 **(C)**, and luminal-basal **(D)**. CI, confidence interval; ER, estrogen receptor; HER2, human epidermal growth factor receptor-2; OR, odds ratio; PR, progesterone receptor; RAI2, retinoic acid-induced 2.

Subsequently, the relation between RAI2 mRNA expression and ER, PR, HER2 status, luminal type, and basal-like BRCA was further evacuated by meta-analysis in GEO datasets. Our results demonstrated that the elevated expression of RAI2 was positively related to BRCA with ER^+^ subtype (pooled OR =4.27, 95% CI: 2.98–6.13, Cochran’s Q test, *p* = 0.000, and *I^2^* = 67.8%; **Figure 2A**) and PR^+^ subtype (pooled OR = 3.16, 95% CI: 2.52–3.96, Cochran’s Q test, *p* = 0.614, and *I^2^* = 0.0%; **Figure 2B**), but it was negatively correlated with BRCA with HER2^+^ subtype (pooled OR =0.68, 95% CI: 0.52–0.89, Cochran’s Q test, *p* = 0.505, and *I^2^* = 0.0%; **Figure 2C**). Besides, there was a positive association between the increased RAI2 expression and luminal subtype of tumors rather than basal-like cancers (pooled OR = 6.95, 95% CI: 5.07–9.51, Cochran’s Q test, *p* = 0.39, and *I^2^* = 4.6%; **Figure 2D**).

### 3.2 RAI2 expression relates to patient survival in BRCA

**Figure 3:**
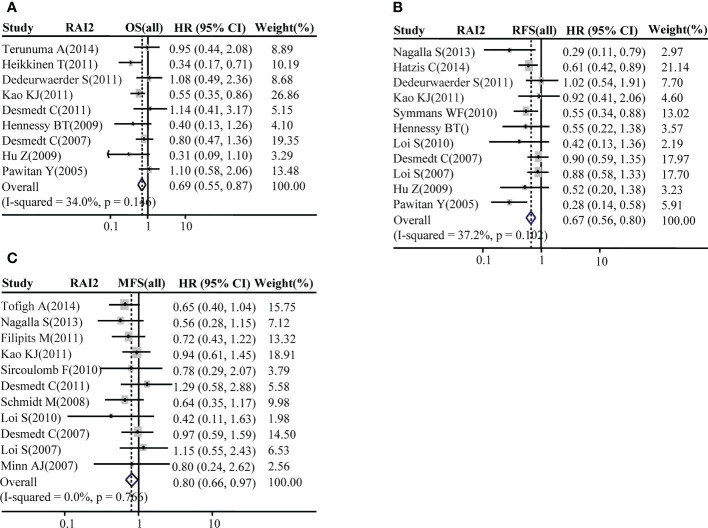
Forest plot for the association of RAI2 mRNA expression with breast cancer survival. Associations between RAI2 mRNA expression with breast cancer OS **(A)**, RFS **(B)**, and MFS **(C)** in all populations of breast cancer. CI, confidence interval; HR, hazard ratio; MFS, metastasis-free survival; OS, overall survival; RFS, recurrence-free survival; RAI2, retinoic acid-induced 2.

The association of RAI2 mRNA expression with survival was evaluated in a total of BRCA patients. Our analysis showed that patients with BRCA with higher RAI2 mRNA level tended to have better OS (pooled OR = 0.69, 95% CI: 0.55–0.87, Cochran’s Q test, *p* = 0.146, and *I^2^* = 34.0%; **Figure 3A**). Moreover, there was a significant association of high RAI2 expression with prolonged RFS (pooled OR = 0.67, 95% CI: 0.56–0.80, Cochran’s Q test, *p* = 0.102, and *I^2^* = 37.2%; **Figure 3B**) and MFS (pooled OR = 0.80, 95% CI: 0.66–0.97, Cochran’s Q test, *p* = 0.766, and *I ^2 =^* 0.0%; **Figure 3C**).

**Figure 4:**
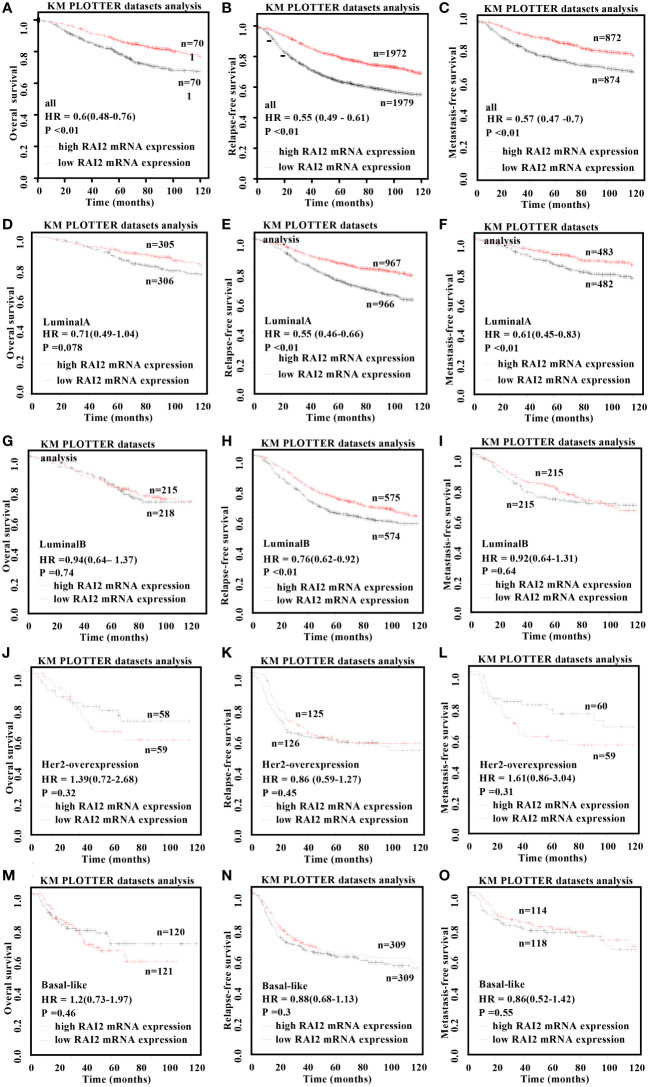
Kaplan–Meier survival curves for the correlation of RAI2 mRNA expression with breast cancer. Overexpression of RAI2 predicted favorable survival in patients with BRCA. The relation of RAI2 expression level in all **(A-C)**, luminal-A **(D-F)**, luminal-B **(G-I)**, her2-overexpression **(J-L)**, and basal-like **(M-O)** breast cancer patients with OS, RFS, and MFS. Her2, human epidermal growth factor receptor 2; MFS, metastasis-free survival; OS, overall survival; RFS, recurrence-free survival; RAI2, retinoic acid-induced 2.

In order to evaluate the prognosis value of RAI2 in distinct molecular subtypes, Kaplan-Meier survival curves were plotted in BRCA patients in **Figure 4**. Our results showed that RAI2 expression was positively associated with the OS (HR = 0.6, 95% CI: 0.48-0.76, *p* < 0.01; **Figure 4A**), RFS (HR = 0.55, 95% CI: 0.49-0.61, *p* < 0.01; **Figure 4B**) and MFS (HR = 0.57, 95% CI: 0.47-0.7, *p* < 0.01; **Figure 4C**) rate in all BRCA patients, it had distinct prognostic value in different subtypes. Although lower RAI2 mRNA expression predicted poor RFS (HR = 0.55, 95% CI: 0.46-0.66, *p* < 0.01; **Figure 4E**) and MFS (HR = 0.61, 95% CI: 0.45-0.83, *p* < 0.01; **Figure 4F**) in patients with luminal A subtype, there was no significant association between RAI2 expression and OS (HR = 0.71, 95% CI: 0.49-1.04, *p* = 0.078; **Figure 4D**) in the patients. In patients with luminal B subtype, lower RAI2 mRNA expression predicted poor RFS (HR = 0.76, 95% CI: 0.62-0.92, *p* < 0.01; **Figure 4H**), whereas there was no significant association between RAI2 and OS (HR = 0.94, 95% CI: 0.64-1.37, *p* = 0.74; **Figure 4G**) and MFS (HR = 0.92, 95% CI: 0.64-1.31, *p* = 0.64; **Figure 4I**). In addition, there was no statistically significant effect of RAI2 on promoting OS (HR=1.39, 95% CI: 0.72-2.68, *p* = 0.32; **Figure 4J**), RFS (HR = 0.86, 95% CI: 0.59-1.27, *p* = 0.45; **Figure 4K**) and MFS (HR=1.61, 95% CI: 0.86-3.04, *p* = 0.31; **Figure 4L**) in patients with HER2-overexpressed subtype, and on prolonging OS (HR=1.2, 95% CI: 0.73-1.97, P = 0.46; **Figure 4M**), RFS (HR = 0.88, 95% CI: 0.68-1.13, P = 0.3; **Figure 4N**) and MFS (HR = 0.86, 95% CI: 0.52-1.42, P = 0.55; **Figure 4O**) in patients with basal-like subtype. In all, the analysis of the RAI2 mRNA level and survival showed that higher RAI2 expression predicted a more favorable prognosis for BRCA patients.

### 3.3 The association of RAI2 expression with six immune infiltrates in BRCA

**Figure 5:**
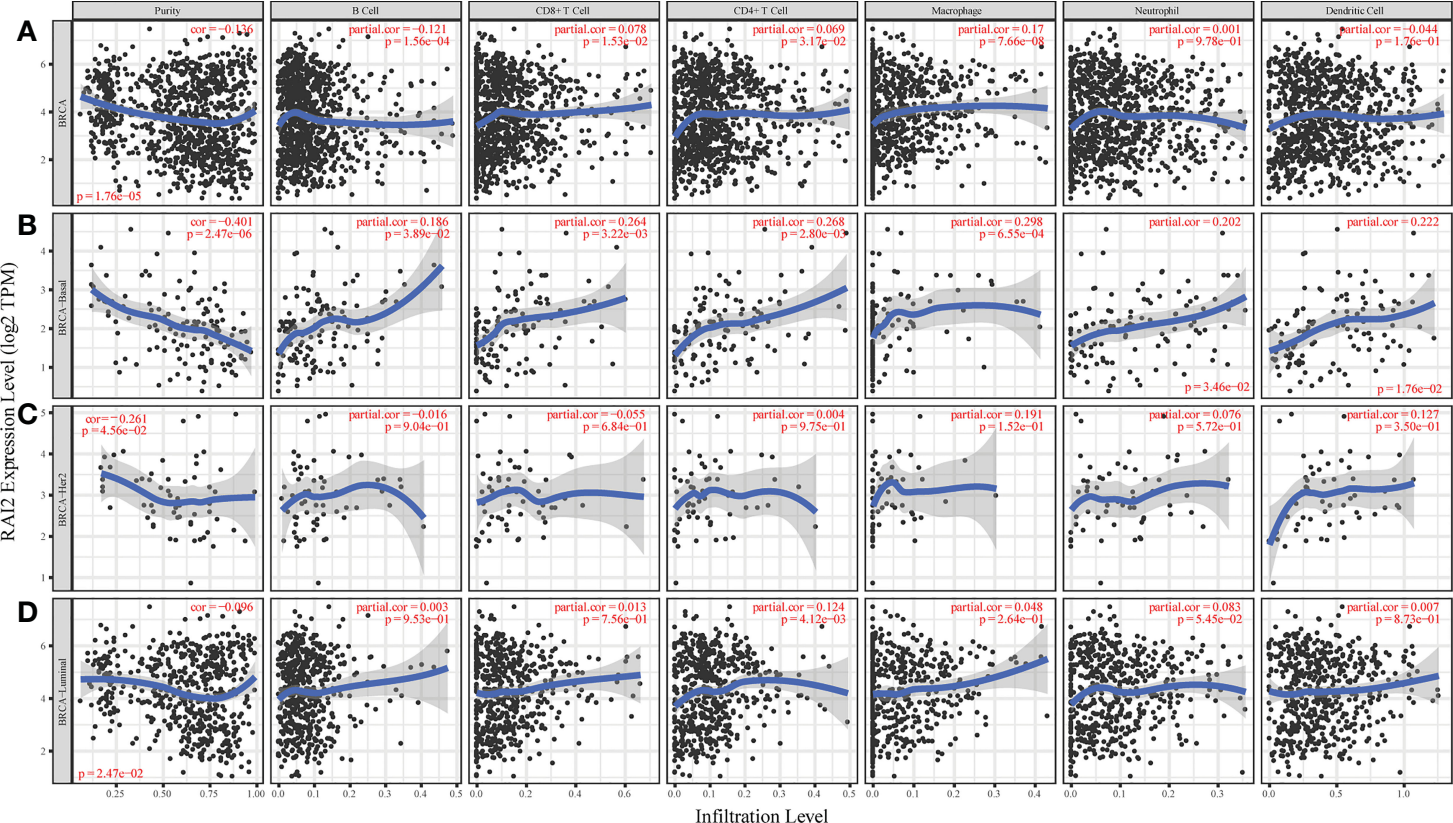
Association of RAI2 expression with immune infiltrating levels in breast cancer. **(A)** RAI2 expression is significantly negatively associated with tumor purity and has a significant positive relation with infiltrating immune cells of B cells, CD8+ T cells, CD4+ T cells, and macrophages, other than neutrophils and dendritic cells. **(B)** RAI2 expression has significant negative correlations with tumor purity and positive infiltrating levels of B cells, CD8+ T cells, CD4+ T, macrophages, neutrophils, and dendritic cells in the BRCA-basal subtype. **(C)** RAI2 expression is negatively related to tumor purity and showed a very weak correlation with infiltrating B cells, CD8+ T cells, CD4+ T, macrophages, neutrophils, and dendritic cells in BRCA-her2 subtype. **(D)** RAI2 expression has negative correlations with tumor purity and positive with infiltrating levels of CD4+ T cells but no significant association with infiltrating level of B cells, CD8+ T cells, macrophages, neutrophils, and dendritic cells in the BRCA-luminal subtype. Her2, human epidermal growth factor receptor 2; RAI2, retinoic acid-induced 2.

Some studies had reported that immune infiltrates were closely related to patient prognosis and could predict the status of sentinel lymph node (22, 23). We utilized the gene module of TIMER datasets to determine the relation of RAI2 expression with tumor-infiltrating lymphocytes for BRCA patients. The analyses results indicated that RAI2 expression was negatively related to tumor purity of BRCA (*r* = -0.136, *p* < 0.001), BRCA-basal (*r* = -0.401, *p* < 0.001), BRCA-HER2 (*r* = -0.136, *p* < 0.001) and BRCA-luminal (*r* = -0.261, *p* < 0.001) subtypes. Furthermore, RAI2 expression level was positively associated with infiltrating levels of CD8+ T cells (*r* = 0.078, *p* = 0.015), CD4+ T cells (*r* = 0.069, *p* = 0.032), and macrophages (*r* = 0.17, *p* < 0.001) in BRCA (**Figure 5A**). In BRAC-basal subtypes (**Figure 5B**), there were a positive association of RAI2 expression within filtrating levels of B cells (*r* = 0.186, *p* = 0.039), CD8+ T cells (*r* = 0.264, *p* = 0.003), CD4+ T cells (*r* = 0.268, *p* = 0.003), macrophages (*r* = 0.298, *p* < 0.001), neutrophils (*r* = 0.202, *p* = 0.035) and DCs (*r* = 0.222, *p* = 0.018). However, there was no relation of RAI2 expression with immune infiltrating levels in BRAC-HER2 subtypes (**Figure 5C**). In addition, RAI2 expression had no association with immune infiltrating levels of B cells, CD8+ T cells, macrophages, neutrophils, and dendritic cells except for CD4+ T cells (*r* = 0.124, *p* = 0.004) in BRAC-luminal subtypes (**Figure 5D**).

### 3.4 Different expression and pathway analysis for DEGs of RAI2^high^ versus RAI2^low^ group

**Figure 6:**
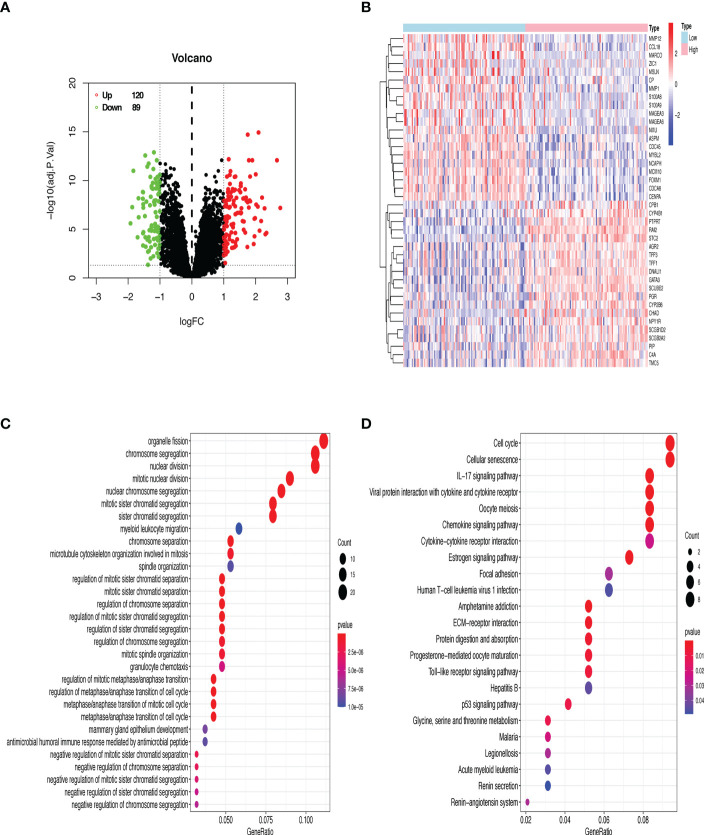
GO and KEGG enrichment analyses of DEGs from the microarray dataset GSE7390. **(A)** 209 DEGs are shown in a volcano plot. 120 upregulated genes are shown in red, and 89 downregulated genes are shown in green. **(B)** Heatmap of the top 20 upregulated and downregulated DEGs. Red denotes upregulated genes, and blue represents downregulated genes. **(C)** The top 30 enriched terms of GO analysis. **(D)** The top 23 enriched terms of the KEGG pathway. The size of dots indicates the count of DEGs enriched under each term.

The datasets of GSE7390 and GSE21653 were respectively divided into two groups (RAI2^high^ and RAI2^low^) by median expression of RAI2 to identify DEGs by R software. Based on the Limma package, a total of 209 DEGs (120 upregulated and 89 downregulated DEGs) in GSE7390 and a total of 182 DEGs (100 upregulated and 82 downregulated DEGs) in GSE21653 were obtained. With GSE7309, a volcano map was employed to show all DEG distribution (**Figure 6A**) and the expression heatmap for 40 genes, comprising the top 20 upregulated and the top 20 downregulated DEGs, was shown in **Figure 6B**. Additionally, the enriched GO terms and KEGG pathways were analyzed for DEGs. In the biological process terms of GO (**Supplemental Table 2A**), our analysis indicated that most of the DEGs in GSE7390 were enriched in “organelle fission” (GO:0048285), “chromosome segregation” (GO:0007059), and “nuclear division” (GO:0000280) (**Figure 6C**). In the KEGG analysis (**Supplemental Table 3A**), our result showed that most of the DEGs in GSE7390 were enriched in the “cell cycle” (hsa04110) and “cellular senescence” (hsa04218) (**Figure 6D**). Using the same analysis strategies, we showed the results of GSE21653 in **Supplemental Figure 1** and **Supplemental Tables 2B**, **3B**. The top 20 upregulated and the top 20 downregulated DEGs in GSE7390 (**Supplemental Figure 2A**) and GSE21653 (**Supplemental Figure 2B**) were respectively analyzed for the correlativity among these genes using R software.

### 3.5 Disease module from the PPI network

**Figure 7:**
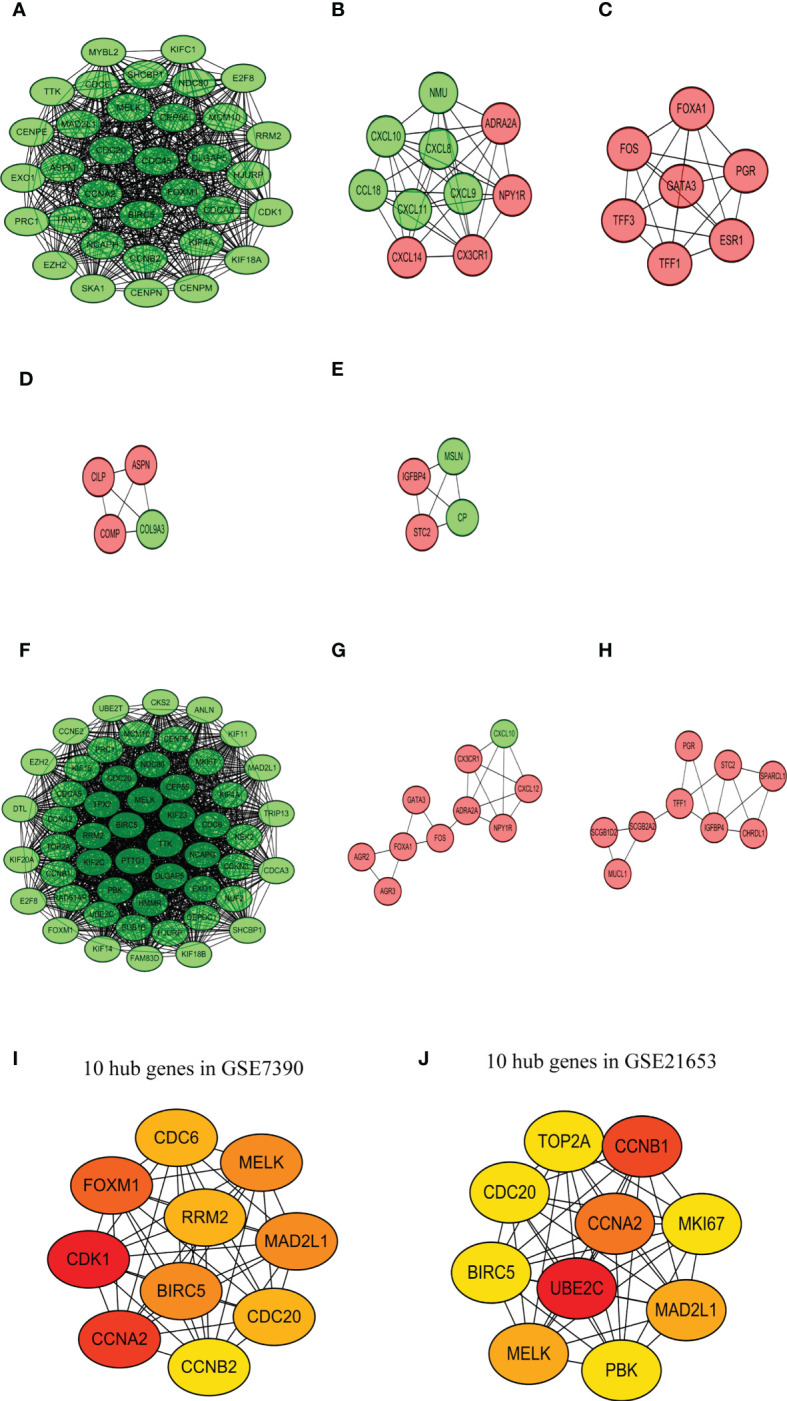
PPI network analysis. Top 5 largest size modules of GSE7390 **(A-E)** and top 3 largest size modules of GSE21653 **(F-H)** from the high-score protein-protein interactive network. 10 hub genes in GSE7390 **(I)** and GSE21653 **(J)**.

The PPIs network of DEGs was screened in the STRING database (24). Genes were included in the network with a medium interaction score (confidence > 0.4) and disconnected nodes were hidden. However, for the two databases, the gene of RAI2 was not shown in the PPI network. Then, the PPI networks were exported to the Cytoscape for visualized results. Most of the up-regulated genes with red and down-regulated genes with blue color were interactional in the PPI visualized network. Analyzing a total of 155 nodes and 860 edges, we discovered the top five largest-size modules of GSE7390 utilizing the MCODE application in Cytoscape (**Figures 7A–E**). KEGG enrichment analysis of the modules indicated that these genes in modules 1–3 were mainly related to “cell cycle” (hsa04110), “viral protein interaction with cytokine and cytokine receptor” (hsa04061), “estrogen signaling pathway” (hsa04915). Analyzing a total of 131 nodes and 1551 edges, we acquired the top three largest size modules of GSE21653 (**Figures 7F–H**). KEGG analysis of the modules showed that these genes in modules 1–2 were implicated with “cell cycle” (hsa04110), and “Viral protein interaction with cytokine and cytokine receptor” (hsa04061). The “cell cycle” (hsa04110) was the common pathway of GSE7390 and GSE21653 in KEGG analysis, with the common genes of CCNA2, CDC20, CDC6, MAD2L1, and TTK. Then, the cytoHubba application in Cytoscape was used to identify the ten hub nodes with the highest degrees and shortest path. The 10 hub genes in GSE7390 contained CDK1, CCNA2, FOXM1, MAD2L1, BIRC5, MELK, CDC20, CDC6, RRM2, CCNB2 (**Figure 7I**; **Supplemental Table 4A**). The ten hub genes in GSE21653 contained UBE2C, CCNB1, CCNA2, MELK, MAD2L1, MKI67, PBK, TOP2A, BIRC5, CDC20 (**Figure 7J**; **Supplemental Table 4B**). In summary, the genes of CCNA2, MAD2L1, MELK, CDC20, and CCNB2 were the common and down-regulated hub genes in the RAI2^high^ group of the two datasets.

### 3.6 The relation of RAI2 expression with several core genes

**Figure 8:**
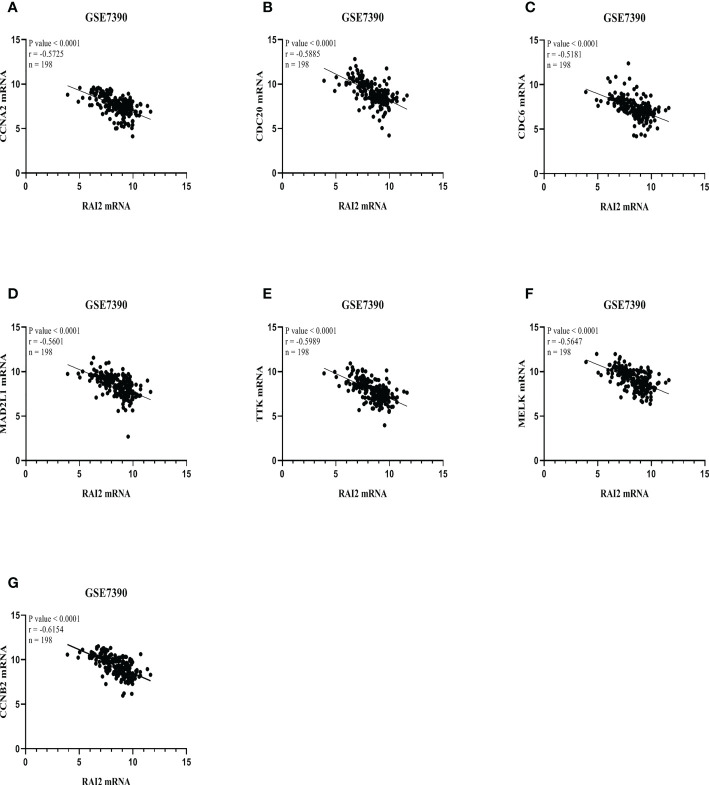
RAI2 expression was associated with several core genes in GSE7390. Association between mRNA expressions of RAI2 with several core genes, including CCNA2 **(A)**, CDC20 **(B)**, CDC6 **(C)**, MAD2L1 **(D)**, TTK **(E)**, MELK **(F)**, and CCNB2 **(G)**. CCNA2, CyclinA2; CCNB2, cyclin B2; CDC6, cell division cycle 6; CDC20, cell division cycle 20; MAD2L1, mitotic arrest deficient 2-like 1; MELK, maternal embryonic leucine zipper kinase; TTK, TTK protein kinase; RAI2, retinoic acid-induced 2.

We performed scatter diagrams and linear regression to show the relations between RAI2 mRNA expression and some core gene mRNA expression in the datasets of GSE7390 and GSE21653. According to the results above, we chose some core genes to estimate their relationship with RAI2. Correlation analysis in GSE7390 demonstrated that RAI2 mRNA expression was negatively correlated with CCAN2 (r = -0.5725, P < 0.001; **Figure 8A**), CDC20 (r = -0.5885, P < 0.001; **Figure 8B**), CDC6 (r = -0.5181, P < 0.001; **Figure 8C**), MAD2L1 (r = -0.5601, P < 0.001; **Figure 8D**), TTK (r = -0.5989, P < 0.001; **Figure 8E**), MELK (r = -0.5647, P < 0.001; **Figure 8F**), CCNB2 (r = -0.6154, P < 0.001; **Figure 8G**) mRNA expression. Additionally, correlation analysis in GSE21653 of RAI2 mRNA expression and these genes further proved the results that RAI2 was negatively correlated with CCAN2 (r = -0.5622, P < 0.001; **Supplemental Figure 3A**), CDC20 (r = -0.5446, P < 0.001; **Supplemental Figure 3B**), CDC6 (r = -0.4985, P < 0.001; **Supplemental Figure 3C**), MAD2L1 (r = -0.4984, P < 0.001; **Supplemental Figure 3D**), TTK (r = -0.5531, P < 0.001; **Supplemental Figure 3E**), MELK (r = -0.5150, P < 0.001; **Supplemental Figure 3F**), CCNB2 (r = -0.5920, P < 0.001; **Supplemental Figure 3G**) mRNA expression.

## 4 Discussion

RAI2 has been proven to suppress early hematogenous dissemination of breast cancer and indicate favorable prognosis by analyzing hundreds of breast cancer patient samples. However, these results need further validation with more patient samples (10, 12). Our results showed that high RAI2 expression could be regarded as an independent and favorable prognostic biomarker for BRCA patients, which predicts tumor invasion and metastasis.

Previous studies found that there was no relation between RAI2 level and lymph node metastasis (10). However, the meta-analysis of 17 GEO datasets in our results showed that lymph node metastasis status had a lower RAI2 mRNA expression level than lymph node non-metastasis status. There was positive relation of RAI2 expression with immune infiltrating levels, including B cells, CD8+ T cells, CD4+ T cells, B cells, and dendritic cells in the BRCA, especially in the BRCA-basal subtype. In addition, our result also showed that RAI2 was positively associated with CX3CR1 and negatively related to CXCL8, respectively. CXCL8 was a well-known chemokine involved in tumorigenesis, tumor progression and immune suppression, and CX3CR1 was regarded as a novel cancer targeted therapeutic strategy due to its immune activation capacity (25, 26). However, the ligands of CX3CR1, CXCL9, 10, and 11 were downregulated by RAI2, which is not consistent with their well-established immunostimulatory function. There may be two explanations for the bias. On the one hand, CXCL9, 10, and 11 promoted immune response by paracrine signal, while tumor-derived autocrine signal will induce tumor cell proliferation and metastasis (26). We could not investigate whether the downregulated expressions of CXCL9, 10, and 11 were induced by paracrine or autocrine axis. On the other hand, divergent immune response occurred in distinct breast cancer subtypes. Generally, any tumor features that oscillated a tumor toward a more aggressive “basal-like” state will typically elicit a stronger immune reaction (27), which is consistent with our result (**Figure 5B**). In the PPI network analysis, we performed the correlation based on the overall BRCA subtypes, which could not visualize and restore the true immune state for each subtype. Therefore, there will be some biases in immune-related chemokines. In general, it was reported that immune infiltrates could predict the status of sentinel lymph nodes and were closely associated with survival (22, 23). Therefore, the high immune infiltrates might contribute to the favorable prognosis in patients with high RAI2 expression.

The results of the functional enrichment of RAI2 mentioned above indicated that RAI2 may be involved in the process of cell proliferation and cell cycle. The common genes in the cell cycle pathway were downregulated in the RAI2^high^ group, including CCNA2, CDC20, CDC6, MAD2L1, and TTK, which suggested that RAI2 expression might regulate the cell cycle by suppressing these gene expressions. Moreover, the common hub genes of DEGs from the two datasets were identified as CCNA2, MAD2L1, MELK, CDC20, and CCNB2.

CCNA2, one of the highly conserved cyclin family, was significantly overexpressed in some cancer cells and had a close relation with tumorigenesis and progression (28). It had been proved that CCNA2 might be implicated in the epithelial-mesenchymal transitions (EMT) and cancer metastasis (29). Furthermore, Gao et al. suggested that high CCNA2 expression predicted poor survival and was also implicated with tamoxifen resistance in ER^+^ mammary cancer patients by bioinformatic analysis (30). Based on TCGA datasets, Tang et al. indicated that CCNB2 was implicated with poor prognosis in BRCA and was significantly up-regulated expressed in the advanced cancer stage (31). CDC20, the cell division regulator, was significantly overexpressed in mammary cancer cells and indicated poor survival (32, 33). Moreover, a preclinical study held that CDC20 in ER^+^ BRCA predicted inadequate response to endocrine therapy and poor survival (34). Cheng et al. firstly proved the association of CDC20 with BRCA metastasis in preclinical research (35). In ER^+^ BRCA, CCNA2, CCNB2, and CDC20 could be inhibited by O6-benzylguanine, an MGMT inhibitor (36). In addition, CDC6 got involved in regulating DNA replication and expressed at a high level in BRCA cells, especially in ER^-^ BRCA, compared with normal breast cells (37). By producing chromosomal instability and aneuploidy, the abnormal expression of MAD2L1 might induce malignant transformation and progression of BRCA (38). TTK, a dual serine/threonine kinase, exerted a vital role in spindle assembly checkpoint signaling and mitotic checkpoint (39, 40). Additionally, by regulating the phosphorylation of p53, CHK2, and MDM2, TTK got involved in DNA damage repair (41–43). Previous studies have shown that TTK was up-regulated in triple-negative BRCA, compared to the other BRCA tissues and healthy cells. Furthermore, TTK regulated EMT and proliferative phenotypes through TGF-β and KLF5 signaling (44, 45). Related research indicated that higher MELK expression was a gene signature in BRCA than in normal tissues (46–48). Giuliano et al. found that MELK expression was related to tumor mitotic activity but was not required for tumor development (49). Deng et al. regarded CCNA2 and MELK as part of potential key genes in the process of mitotic cell cycle and EMT pathway for the progression of BRCA by bioinformatic analysis (50).

From the results above, we suggested that RAI2 might get involved in inhibiting the proliferation, invasion, and metastasis of BRCA cells and improving the immune infiltrating levels in BRAC. Moreover, in the development process of BRCA, the lower expression of RAI2 was implicated in the higher expression of CCNA2, CCNB2, CDC20, CDC6, MAD2L1, TTK, and MELK. To discover the role of RAI2 in BRCA, the specific biological mechanisms of the interaction of these genes need further research.

## 5 Conclusion

Taken together, we estimated the predictive value of RAI2 for BRCA patients. RAI2 could be conceived as a promising prognostic biomarker in each subtype of BRCA. Therefore, our analyses might support RAI2 as the potential prognostic biomarker and contribute to further research of RAI2 as the candidate for therapy target in BRCA diagnosis and treatment.

## Data availability statement

Publicly available datasets in this study can be found in TIMER https://cistrome.shinyapps.io/timer/) and GEO (https://www.ncbi.nlm.nih.gov/geo/).

## Author contributions

YJ prepared the figures and drafted the manuscript. SL collected data and revised the manuscript and participated in the discussion. JG, KZ, and YX participated in the discussion. All authors contributed to the article and approved the submitted version.

## Supplementary material

Supplementary Figure 1 GO and KEGG enrichment analyses of DEGs from the microarray dataset GSE21653. **(A)** 182 DEGs are shown in a volcano plot. 100 upregulated genes are shown in red, and 82 downregulated genes are shown in green. **(B)** Heatmap of the top 20 upregulated and downregulated DEGs. Red denotes upregulated genes, and blue represents downregulated genes. **(A)** The top 30 enriched terms of GO analysis. **(B)** The top 9 enriched terms of the KEGG pathway. The size of dots indicates the count of DEGs enriched under each term.

Supplementary Figure 2 The correlativity of the top 20 upregulated and the top 20 downregulated DEGs in GSE7390 **(A)** and GSE21653 **(B)**.

Supplementary Figure 3 RAI2 expression was associated with several core genes in GSE21653. Association between mRNA expressions of RAI2 with several core genes, including CCNA2 **(A)**, CDC20 **(B)**, CDC6 **(C)**, MAD2L1 **(D)**, TTK **(E)**, MELK **(F)**, and CCNB2 **(G)**. CCNA2, CyclinA2; CCNB2, cyclin B2; CDC6, cell division cycle 6; CDC20, cell division cycle 20; MAD2L1, mitotic arrest deficient 2-like 1; MELK, maternal embryonic leucine zipper kinase; TTK, TTK protein kinase; RAI2, retinoic acid-induced 2.
