# Supplementary material for: Comprehensive analysis of the expression and prognosis for RAI2: A promising biomarker in breast cancer
Source: Front Oncol. 2023 Mar 29;13:1134149. doi: 10.3389/fonc.2023.1134149 (PMC10090471; doi:10.3389/fonc.2023.1134149)
Supplement: Supplementary file 2 [file Table_2.docx]

Table 2: Gene Ontology analysis of DEGs in GSE7390 (A) and GSE21653 (B) with breast cancer.

| A, GSE7390 | | |  |  |
| --- | --- | --- | --- | --- |
| Category | GO ID | Gene function |  |  |
| BP | GO:0048285 | organelle fission | Gene count | P-value |
| BP | GO:0007059 | chromosome segregation | 21 | 6.05×10^-9^ |
| BP | GO:0000280 | nuclear division | 20 | 1.00×10^-10^ |
| BP | GO:0140014 | mitotic nuclear division | 20 | 6.21×10^-9^ |
| BP | GO:0050900 | leukocyte migration | 17 | 1.65×10^-9^ |
| CC | GO:0062023 | collagen-containing extracellular matrix | 17 | 1.35×10^-5^ |
| CC | GO:0005819 | spindle | 18 | 9.18×10^-8^ |
| CC | GO:0098687 | chromosomal region | 17 | 5.09×10^-8^ |
| CC | GO:0000775 | chromosome, centromeric region | 14 | 8.23×10^-6^ |
| CC | GO:0005874 | microtubule | 12 | 4.13×10^-7^ |
| MF | GO:0001228 | DNA-binding transcription activator activity, RNA polymerase II-specific | 11 | 2.5×10^-3^ |
| MF | GO:0008017 | microtubule binding | 14 | 2.5×10^-4^ |
| MF | GO:0015631 | tubulin binding | 12 | 1.24×10^-5^ |
| MF | GO:0001664 | G protein-coupled receptor binding | 12 | 2.5×10^-4^ |
| MF | GO:0048018 | receptor ligand activity | 11 | 2.0×10^-4^ |
|  |  |  | 11 | 0.013 |
| B, GSE21653 | | | | |
| Category | | GO ID | Gene function |  |
| BP | GO:0000280 | nuclear division | Gene count | P-value |
| BP | GO:0048285 | organelle fission | 31 | 4.91×10^-21^ |
| BP | GO:0007059 | chromosome segregation | 31 | 8.70×10^-20^ |
| BP | GO:0140014 | mitotic nuclear division | 29 | 9.02×10^-22^ |
| BP | GO:0098813 | nuclear chromosome segregation | 27 | 9.92×10^-22^ |
| CC | GO:0005819 | spindle | 25 | 1.98×10^-19^ |
| CC | GO:0000793 | condensed chromosome | 21 | 7.20×10^-13^ |
| CC | GO:0062023 | collagen-containing extracellular matrix | 16 | 3.66×10^-11^ |
| CC | GO:0098687 | chromosomal region | 16 | 1.94×10^-7^ |
| CC | GO:0000775 | chromosome, centromeric region | 15 | 1.58×10^-7^ |
| MF | GO:0008017 | microtubule binding | 14 | 5.61×10^-10^ |
| MF | GO:0015631 | tubulin binding | 16 | 2.82×10^-10^ |
| MF | GO:0016887 | ATPase activity | 16 | 2.49×10^-8^ |
| MF | GO:0003777 | microtubule motor activity | 10 | 1.55×10^-3^ |
| MF | GO:0003774 | motor activity | 9 | 3.59×10^-8^ |
|  |  |  |  |  |
