# Supplementary material for: Comprehensive analysis of the expression and prognosis for RAI2: A promising biomarker in breast cancer
Source: Front Oncol. 2023 Mar 29;13:1134149. doi: 10.3389/fonc.2023.1134149 (PMC10090471; doi:10.3389/fonc.2023.1134149)
Supplement: Supplementary file 3 [file Table_3.docx]

Table 3: Kyoto Encyclopedia of Genes and Genomes pathway analysis of DEGs in GSE7390 (A) and GSE21653 (B) with breast cancer.

| A, GSE7390 |  |  |  |  |  |  |
| --- | --- | --- | --- | --- | --- | --- |
| Pathway ID | Name | Count | P-value | Genes |  |  |
| hsa04110 | Cell cycle | 9 | 1.55E-05 | CCNB2/CDC20/TTK/CCNA2/MAD2L1/CCNE1/CDK1/CDC6/CDC45 | | |
| hsa04218 | Cellular senescence | 9 | 0.00011554 | CCNB2/FOXM1/CCNA2/CCNE1/CACNA1D/MYBL2/CDK1/CXCL8/CALML5 | | |
| hsa04657 | IL-17 signaling pathway | 8 | 1.48E-05 | S100A8/MMP1/CXCL10/S100A9/FOS/CXCL8/FOSB/S100A7 | | |
| hsa04061 | Viral protein interaction with cytokine and cytokine receptor | 8 | 2.33E-05 | CX3CR1/IL6ST/CCL18/CXCL14/CXCL10/CXCL8/CXCL9/CXCL11 | | |
| hsa04114 | Oocyte meiosis | 8 | 0.00013607 | CCNB2/CDC20/MAD2L1/AR/CCNE1/CDK1/PGR/CALML5 | | |
|  |  |  |  |  |  |  |
| B, GSE21653 |  |  |  |  |  |  |
| Pathway ID | Name | Count | P-value | Genes |  |  |
| hsa04110 | Cell cycle | 9 | 9.01E-07 | PTTG1/CCNA2/BUB1B/CDC20/TTK/CDC6/MAD2L1/CCNE2/CCNB1 | | |
| hsa04114 | Oocyte meiosis | 8 | 1.19E-05 | PTTG1/CDC20/MAD2L1/AR/CCNE2/CCNB1/PGR/CALML5 | | |
| hsa04218 | Cellular senescence | 5 | 0.01203278 | CCNA2/FOXM1/CCNE2/CCNB1/CALML5 | | |
| hsa04061 | Viral protein interaction with cytokine and cytokine receptor | 4 | 0.01069609 | CX3CR1/CXCL12/CXCL14/CXCL10 | | |
| hsa04115 | p53 signaling pathway | 3 | 0.02412344 | CCNE2/CCNB1/RRM2 | |  |
