# Supplementary material for: Comprehensive analysis of the expression and prognosis for RAI2: A promising biomarker in breast cancer
Source: Front Oncol. 2023 Mar 29;13:1134149. doi: 10.3389/fonc.2023.1134149 (PMC10090471; doi:10.3389/fonc.2023.1134149)
Supplement: Supplementary file 4 [file Table_4.docx]

Table 4: Hub genes and rank of degrees in GSE7390 (A) and GSE21653 (B) with breast cancer.

| A, GSE7390 |  |  |
| --- | --- | --- |
| Gene symbol | Full name | Degree |
| CDK1 | Cyclin dependent kinase 1 | 42 |
| CCNA2 | Cyclin A2 | 41 |
| FOXM1 | Forkhead box M1 | 39 |
| MAD2L1 | MAD2 mitotic arrest deficient-like 1 | 36 |
| BIRC5 | Baculoviral initiator of apoptosis repeat containing 5 | 36 |
| MELK | Maternal embryonic leucine zipper kinase | 36 |
| CDC20 | Cell division cycle 20 | 35 |
| CDC6 | Cell division cycle 6 | 35 |
| RRM2 | Ribonucleotide reductase regulatory subunit M2 | 35 |
| CCNB2 | Cyclin B2 | 34 |
|  |  |  |
| B, GSE21653 |  |  |
| Gene symbol | Full name | Degree |
| UBE2C | Ubiquitin conjugating enzyme E2 C | 59 |
| CCNB1 | Cyclin B2 | 58 |
| CCNA2 | Cyclin A2 | 57 |
| MELK | Maternal embryonic leucine zipper kinase | 56 |
| MAD2L1 | MAD2 mitotic arrest deficient-like 1 | 56 |
| MKI67 | Marker of proliferation Ki-67 | 55 |
| PBK | PDZ binding kinase | 55 |
| TOP2A | DNA topoisomerase II alpha | 55 |
| BIRC5 | Baculoviral initiator of apoptosis repeat containing 5 | 55 |
| CDC20 | Cell division cycle 20 | 55 |
