# Supplementary figures and images for: Comprehensive analysis of the expression and prognosis for RAI2: A promising biomarker in breast cancer
Source: Front Oncol. 2023 Mar 29;13:1134149. doi: 10.3389/fonc.2023.1134149 (PMC10090471; doi:10.3389/fonc.2023.1134149)

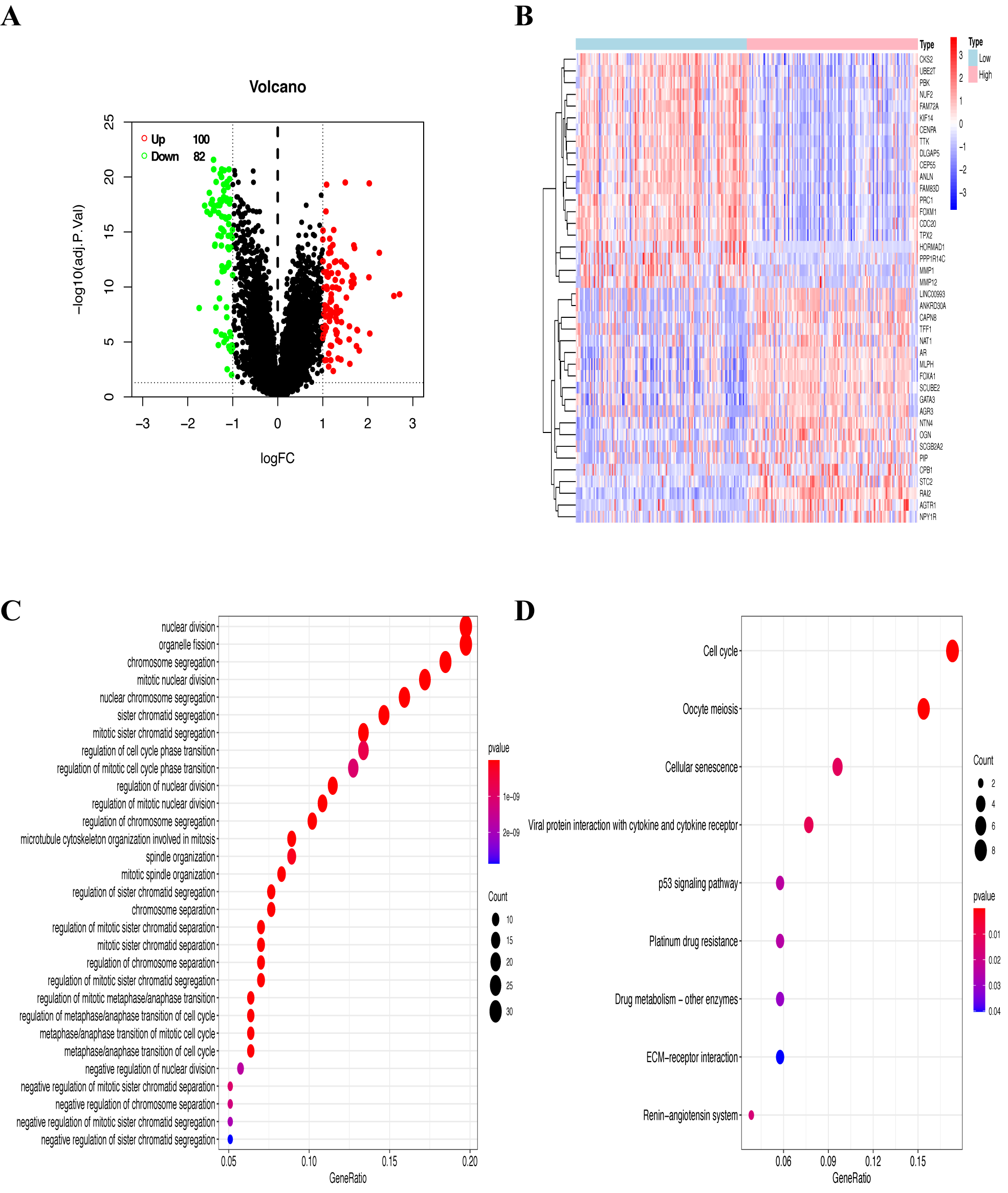

Supplement: Supplementary Figure 1 — GO and KEGG enrichment analyses of DEGs from the microarray dataset GSE21653. (A) 182 DEGs are shown in a volcano plot. 100 upregulated genes are shown in red, and 82 downregulated genes are shown in green. (B) Heatmap of the top 20 upregulated and downregulated DEGs. Red denotes upregulated genes, and blue represents downregulated genes. (A) The top 30 enriched terms of GO analysis. (B) The top 9 enriched terms of the KEGG pathway. The size of dots indicates the count of DEGs enriched under each term. [file Image_1.tif]

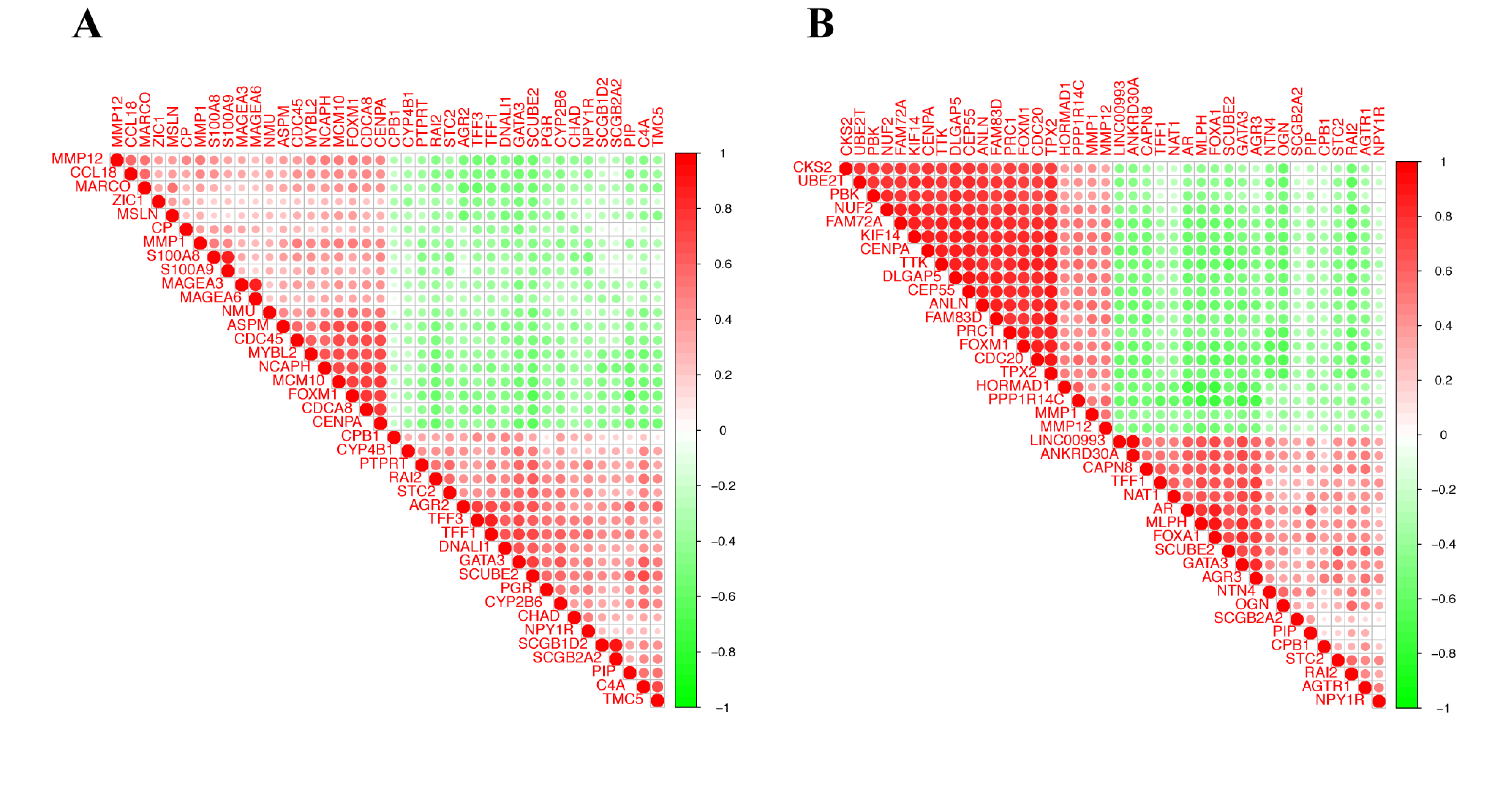

Supplement: Supplementary Figure 2 — The correlativity of the top 20 upregulated and the top 20 downregulated DEGs in GSE7390 (A) and GSE21653 (B). [file Image_2.tif]

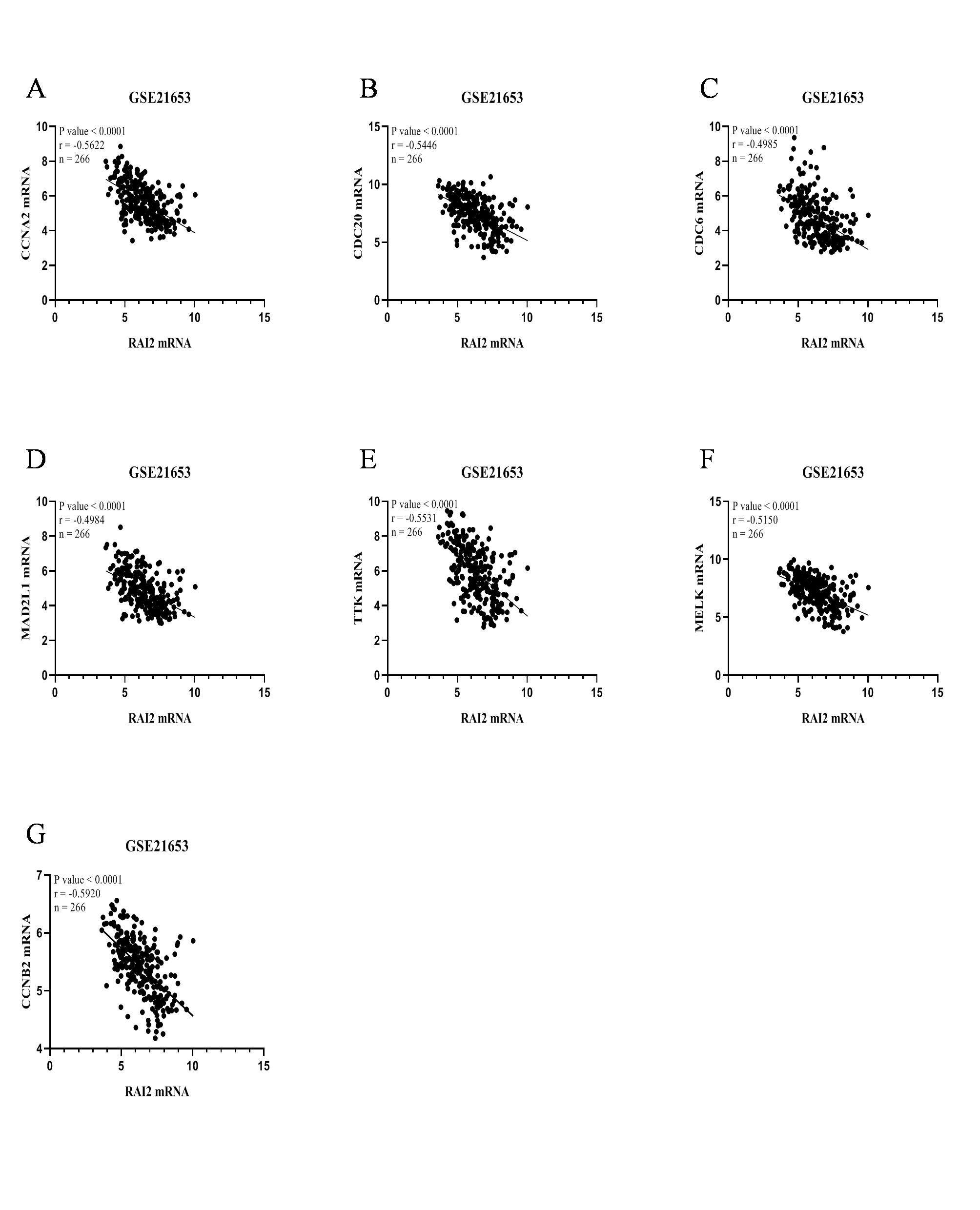

Supplement: Supplementary Figure 3 — RAI2 expression was associated with several core genes in GSE21653. Association between mRNA expressions of RAI2 with several core genes, including CCNA2 (A), CDC20 (B), CDC6 (C), MAD2L1 (D), TTK (E), MELK (F), and CCNB2 (G). CCNA2, CyclinA2; CCNB2, cyclin B2; CDC6, cell division cycle 6; CDC20, cell division cycle 20; MAD2L1, mitotic arrest deficient 2-like 1; MELK, maternal embryonic leucine zipper kinase; TTK, TTK protein kinase; RAI2, retinoic acid-induced 2. [file Image_3.tif]
